# Reply: ‘The importance of study design in the application of artificial intelligence methods in medicine’

**DOI:** 10.1038/s41746-019-0175-0

**Published:** 2019-10-18

**Authors:** Kunal Nagpal, Yun Liu, Po-Hsuan Cameron Chen, Martin C. Stumpe, Craig H. Mermel

**Affiliations:** 1 Google Health, Palo Alto, CA USA; 2 Present Address: Tempus Labs Inc, Chicago, IL USA

**Keywords:** Pathology, Prostate cancer

We thank Eklund and colleagues for their interest and thoughtful comments on our study^1^ and its potential impact on prostate cancer patients. We wholeheartedly agree with Eklund et al. that careful validation of artificial intelligence (AI) algorithms—whether our’s or other’s—will be required to assess their true clinical value.

For the benefit of the broader readership, we would like to clarify that Gleason scoring is used at two distinct stages in prostate cancer diagnosis: first during the biopsy stage (where small samples of prostate tissue are extracted), and second, for a subset of patients during radical prostatectomy (RP, surgical removal of the entire prostate). During biopsy, the Gleason score has a critical role in guiding treatment decisions, including whether to undergo the RP procedure, whereas during the RP stage, the Gleason score is used for prognostication and to determine the need for further adjuvant therapy post surgery.

Our study focused on the Gleason scoring of RP specimens for several reasons outlined in the manuscript, including the larger size of the available tissue leading to bigger development and validation data sets, more context for pathologists and specialists to determine the reference standard grade, and a link with outcomes that is less confounded by diverging treatment pathways than for needle core biopsies. As Eklund and colleagues alluded to, we have not validated our algorithm on biopsies (which has a bigger role in therapy decisions including the decision to undergo RP) in this study and have instead described the application to biopsies as future work.

A second point raised by Eklund and colleagues is the potential confounding of the outcomes analysis owing to the pathologist’s grades being used to determine therapy. To be clear, both the general and genitourinary (GU)-specialist pathologists graded these specimens solely as part of this study and these grades did not affect the original care pathway for any of the patients whose specimens were included in this study. Furthermore, our study’s primary analyses (Figs 2–4) focused on comparing the Gleason scoring by pathologists to the deep-learning system using a reference standard derived from GU-specialist pathologists to enable a direct head-to-head comparison of grading accuracy.

Figure 5 indeed attempts to assess the correlation of the deep learning system’s Gleason scoring with clinical outcomes. We acknowledge that retrospective outcomes analyses such as these may be confounded by uncontrolled treatment variables including the use of post-surgical adjuvant therapy, and that these analyses would have been improved by controlling for such effects; unfortunately, further treatment information was not available for the specimens used in this analysis. Both because of the potential treatment confounding and the limited sample size, we emphasized the trend towards better prognostication in that analysis rather than more conclusive statements. We stand by our original conclusion that the paper demonstrates “improved Gleason scoring” on the basis of the comprehensive, unconfounded analysis presented in Figs 2–4 of the manuscript.

To conclude, we agree with Eklund and colleague’s broader comments about the importance of careful validation of AI algorithms. Although we believe the results in this study point to the great potential for AI-based tools to improve the accuracy and reliability of Gleason grading and subsequent treatment decisions for men with prostate cancer, this is but the first step on the road toward clinical adoption. That road must include randomized, prospective studies to avoid the potential for confounding that exists in any retrospective outcomes analysis.
